# Cellular Mechanisms of Singlet Oxygen in Photodynamic Therapy

**DOI:** 10.3390/ijms242316890

**Published:** 2023-11-29

**Authors:** Maria Przygoda, Dorota Bartusik-Aebisher, Klaudia Dynarowicz, Grzegorz Cieślar, Aleksandra Kawczyk-Krupka, David Aebisher

**Affiliations:** 1 Students English Division Science Club, Medical College of The University of Rzeszów, 35-315 Rzeszów, Poland; maria.przygoda@interia.pl; 2 Department of Biochemistry and General Chemistry, Medical College of The University of Rzeszów, 35-959 Rzeszów, Poland; dbartusikaebisher@ur.edu.pl; 3 Center for Innovative Research in Medical and Natural Sciences, Medical College of The University of Rzeszów, 35-310 Rzeszów, Poland; kdynarowicz@ur.edu.pl; 4 Department of Internal Medicine, Angiology and Physical Medicine, Center for Laser Diagnostics and Therapy, Medical University of Silesia in Katowice, Batorego 15 Street, 41-902 Bytom, Poland; cieslar1@tlen.pl; 5 Department of Photomedicine and Physical Chemistry, Medical College of The University of Rzeszów, 35-959 Rzeszów, Poland

**Keywords:** photodynamic therapy (PDT), reactive oxygen species (ROS), anticancer properties

## Abstract

In this review, we delve into the realm of photodynamic therapy (PDT), an established method for combating cancer. The foundation of PDT lies in the activation of a photosensitizing agent using specific wavelengths of light, resulting in the generation of reactive oxygen species (ROS), notably singlet oxygen (^1^O_2_). We explore PDT’s intricacies, emphasizing its precise targeting of cancer cells while sparing healthy tissue. We examine the pivotal role of singlet oxygen in initiating apoptosis and other cell death pathways, highlighting its potential for minimally invasive cancer treatment. Additionally, we delve into the complex interplay of cellular components, including catalase and NOX1, in defending cancer cells against PDT-induced oxidative and nitrative stress. We unveil an intriguing auto-amplifying mechanism involving secondary singlet oxygen production and catalase inactivation, offering promising avenues for enhancing PDT’s effectiveness. In conclusion, our review unravels PDT’s inner workings and underscores the importance of selective illumination and photosensitizer properties for achieving precision in cancer therapy. The exploration of cellular responses and interactions reveals opportunities for refining and optimizing PDT, which holds significant potential in the ongoing fight against cancer.

## 1. Introduction

### 1.1. Basics Operation of PDT

Photodynamic therapy (PDT) is a well-researched anticancer therapy method whose clinical use dates back to 1960 [1]. The action of PDT is based on the use of a photosensitizing agent (PS) which, after its activation with light of a specific wavelength in the presence of molecular oxygen [2], results in selective tissue damage by having a cytotoxic effect on cancer cells. The most frequently described PSs include Photofrin^®^, 5-aminolevulinic acid (5-ALA), and 5-aminolevulinic acid hexyl (HLA). Photosensitizers are administered topically or systemically (intraveneously) and then, during the drug–light interval (the time elapsed between PS administration and tissue illumination), they are allowed to accumulate in cancerous tissues prior to light exposure. Under the influence of light, energy is transferred to molecular oxygen, resulting in the formation of reactive oxygen species (ROS), including singlet oxygen (^1^O_2_), superoxide radical (O_2_^−•^), hydroxyl radical (HO•), and hydrogen peroxide (H_2_O_2_) [2]. This leads to the initiation of a cascade of biochemical events that in turn lead to the damaging and death of cancer cells [3].

**Figure 1:**
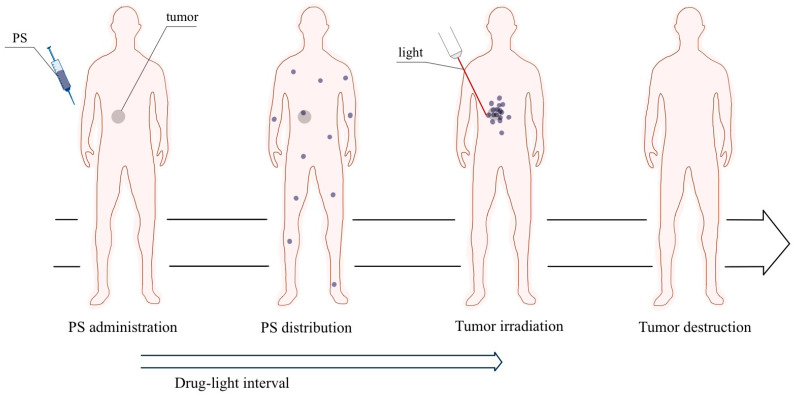
Operation of PDT illustrated schematically. The photosensitizer is administered intravenously and PS accumulates in tumor tissues. Light of a specific wavelength activates PS to produce ROS. This process leads to the initiation of a cascade of biochemical events that lead to damage and death of cancer cells.

Photodynamic therapy leads to selective tissue destruction caused by ROS generated during the photochemical reactions, which are short-lived and highly reactive. They primarily affect cells that uptake PS, leading to local destruction of the target tissue (Figure 1). One of the significant advantages of PDT is the ability to selectively target abnormal cells with light while minimizing the damage to surrounding healthy tissue. This feature makes it a precise and minimally invasive treatment compared to other cancer treatment methods. Photodynamic therapy also stimulates inflammatory and anti-inflammatory immune responses that inhibit or enhance the destruction of target cells.

This review provides a description of the involvement of reactive oxygen/nitrogen species (ROS/RNS) in cancer progression and a description of the diverse functions of membrane-bound catalase in cancer cells, particularly its central role in the regulation of ROS-/RNS-mediated apoptosis signaling. This review also highlights how the introduction of exogenous singlet oxygen or the modulation of intracellular NO levels can inactivate or inhibit membrane-bound catalase, leading to the appearance of secondary singlet oxygen and the establishment of a self-amplifying singlet oxygen production system. This system then causes the inactivation of catalase and the reactivation of intercellular signaling, which induces apoptosis.

This review discusses the role of reactive ROS/RNS in cancer cells during tumor growth and PDT treatment. We draw attention to the diverse biological activity of ROS/RNS associated with cancer cell membranes. In light of these mechanisms, we present PDT from the angle of the generation and subsequent cellular action of singlet oxygen.

### 1.2. Main Components of PDT

#### 1.2.1. Oxygen

Molecular oxygen is one of the three critical components of the PDT mechanism and plays a key role in the production of ROS. The presence of oxygen significantly affects PDT, and its concentration can vary significantly between different tumors and even in different areas of the same tumor, depending on the vascular density. Particularly in deeper solid tumors, characterized by an oxygen-deprived microenvironment, oxygen deficiency is a limiting factor. High-intensity luminous flux irradiation may transiently deplete local oxygen levels, causing a halt in ROS production and reduced treatment effectiveness. Oxygen depletion occurs when the rate of oxygen consumption in the photodynamic reaction exceeds the rate of oxygen diffusion into the irradiated area. Furthermore, PDT may lead to tumor vascular occlusion, reducing the blood flow to the tumor tissue and exacerbating hypoxia [4,5]. An immediate challenge in PDT is to measure tissue oxygen levels in real time before and during treatment, which could optimize therapeutic outcomes by adjusting the light fluence ratio (increasing the exposure time to maintain the total light dose) or using fractional light doses. Various sensors have been used to monitor oxygen levels in biological media, often in combination with imaging agents. However, reports on the integration of these imaging agents with photosensitizers are limited [6]. Several strategies have been explored to increase oxygen availability in tumors. One indirect approach involves increasing the intracellular oxygen levels in cancer cells by using catalase enzymes to break down hydrogen peroxide into oxygen. The direct introduction of oxygen into tumors is achieved through the use of oxygen carriers such as perfluorocarbons and hemoglobin, which are commonly used to counteract tumor hypoxia during PDT procedures [7]. In cells, due to the proton pumps in the membrane, peroxynitrite is protonated and the resultant peroxynitrous acid generates ^•^NO_2_ and hydroxyl radicals according to the following equations [8]:

ONOO^−^ + H^+^ → ONOOH

ONOOH → ^•^NO_2_ + ^•^OH

The interaction between H_2_O_2_ and hydroxyl radicals leads to the formation of hydroperoxide radicals that seem to react with NOX1-derived superoxide anions and form singlet oxygen [9,10,11]:

^•^OH + H_2_O_2_ → HO_2_^•^ + H_2_O

HO_2_^•^ + O_2_^•−^ + H^+^ → H_2_O_2_ + ^1^O_2_

Furthermore, singlet oxygen has been shown to inactivate the reaction of catalase with histidine in the active center of the enzyme [12,13,14].

Singlet oxygen has a lifetime from nanoseconds to microseconds, with a diffusion distance of ^1^O_2_ in the nanometer range.

Thirty-seven years ago, Eisenberg et al. [15] reported a Pyrex-tube with an adsorbed rose bengal PS that generated singlet oxygen upon irradiation when oxygen gas was passed through it. Singlet oxygen exited the distal end of the tube. Singlet oxygen was transported through a space with a width of ~1.5 mm, which corresponded to a lifetime of 54 ms. Despite the novelty, the Eisenberg Pyrex-tube method and other gas–solid methods to generate external ^1^O_2_ are not compatible with in vivo photodynamic therapy because of high oxygen gas flow rates, which would cool the biological media and evaporate water from it.

The consensus in the photodynamic therapy literature is that the singlet oxygen diffusion distance in cells is shorter than the diameter of a typical intracellular organelle [16]. Recent papers by Niedre et al., and Chen et al. [17,18] and Kanofsky [19,20] and Skovsen et al., and Zebger et al. [21,22] report the detection of singlet oxygen luminescence in cells and tissues. The lifetime of singlet oxygen in pure water is 3.1 µs, in an intracellular environment it is most likely less than 3 µs, and in D_2_O it is 68 µs [23]. The square of the distance (*d*) that an oxygen molecule travels during a time (*t*) with the diffusion coefficient (D_o_) is given by the equation *d*^2^ = 6D_o_*t* [24]. The translational diffusion distance of singlet oxygen cellular environments is ~100–150 nm and 20 nm inside cells showing 0.04 µs singlet oxygen lifetimes in skin and the liver of living rats [25]. 

#### 1.2.2. Photosensitizers

Photosensitizers are an integral element used to achieve the therapeutic effects of PDT. Their desired properties are selectivity and accumulation in cancer cells, a low dark toxicity, light absorption peaks between approximately 600 nm and 800 nm, amphiphilicity, a high quantum yield of singlet oxygen generation, and rapid clearance from the patient’s body [26]. Currently, the most frequently used PS preparations in clinical practice are derivatives of porphyrins, phthalocyanines, and chlorins [27].

Photosensitizers can be classified into three generations. First-generation PSs are derivatives of hematoporphyrin (porfimer sodium salt and HpD). The second-generation PSs include 5-aminolevulinic acid (5-ALA) and its esters, benzoporphyrin derivative (BPD), temoporfin (mTHPC, Foscan), tin etiopururin (SnET2), sodium taloporfin (LS11), and lutetium texaphyrin. Second-generation PSs are distinguished by a strong absorption of red light. The third-generation PSs are compounds combining PS with biomolecules or carriers with an affinity for cancer cells. Third-generation PSs show an increased selectivity towards tumor tissue, thanks to the combination of PS with targeting molecules, which distinguishes them from earlier generations of PSs [28,29]. Such carriers include antibodies, proteins, and carbohydrates, as well as carriers such as silica nanoparticles, gold, quantum dots, and others [28,30,31]. The first generation of PSs, such as HpD, have a rich clinical history spanning over 30 years [32]. Photofrin^®^, the first clinically approved PS, has been used to treat a variety of cancers, including lung, bladder, esophageal, and brain cancers [33,34]. Despite their wide application, first-generation PSs have several drawbacks, including low chemical purities and effective activation only at wavelengths below 640 nm, which limits the depth of activating light tissue penetration. Additionally, their extended half-life causes skin hypersensitivity to ambient light which lasts for several weeks, requiring patients to stay inside for up to six weeks. To overcome these limitations, second-generation photosensitizers appeared in the late 1980s [29,32]. Second-generation PSs include pure synthetic compounds with aromatic macrocycles, such as porphyrins, benzoporphyrins, chlorins, bacteriochlorins, and phthalocyanines [35]. Notable examples of clinically approved or ongoing PS trials in this category include temoporfin (Foscan^®^), motexafine lutetium (Lutex^®^), palladium bacteriopheophorbide (soluble Tookad^®^), ethyl tin etiopurpurin (Purlytin^®^), verteporfin (Visudyne^®^), and talapor-fin (Laserphyrin^®^) [33]. These porphyrinoid compounds provide improved tumor specificity and activation in deeper tissue due to absorptions in the 650–800 nm range.

Second-generation PSs are also characterized by faster elimination from the body, which results in fewer side effects and a shorter time for the patient to avoid sunlight (usually less than two weeks). However, their significant disadvantage is poor solubility in water, which leads to aggregation under physiological conditions and reduces the efficiency of the production of ROS. Their hydrophobic nature also creates challenges in the case of intravenous administration, which necessitates a search for new methods of drug delivery [29]. In response, the development of third-generation PSs has focused on the synthesis of structures with greater affinities for target cells [29]. These PSs often consist of a second-generation PS or a photoactivated drug integrated with biodegradable/biocompatible nanoparticles (NPs). This approach increases PS stability, hydrophilicity, pharmacokinetics, pharmacodynamics, and in vivo biodistribution, as well as reducing undesirable side effects and limiting dark toxicity [36,37]. Although significant progress has been made over the past decade, third-generation PSs are still under development, and none are clinically approved as of yet [38]. The challenges associated with PS administration hinder the widespread clinical application of photodynamic therapy, highlighting the urgent need for improved drug delivery systems to increase the therapy’s effectiveness [29].

Light absorbed by molecules causes photochemical changes. The PS molar absorption coefficient obeys the Beer–Lambert law: absorbance = −log10(transmittance%). The wavelength of the absorbance peak and its shape depend on the concentration, pH, solvent, etc. Photosensitizers are excited to the singlet state upon irradiation. The singlet state can relax and generate heat, fluoresce, or intersystem cross (ISC) to the excited triplet state. The triplet state PS transfers energy to triplet ground-state oxygen (^3^O_2_) to yield ^1^O_2_. Photosensitizers are also used for the fluorescence imaging of tumor margins (e.g., bladder and glioma) [39].

Minor structural changes influence the photosensitizing abilities of visible-light-absorbing molecules, for example the “heavy atom effect,” in which atoms with a higher atomic number promote the formation of excited triplets. Halogen substitution affords ISC (e.g., bromination of rhodamine 123). Replacing oxygen with sulfur or selenium increases the production of singlet oxygen (e.g., merocyanine 540). At high concentrations, photosensitizer aggregation limits transition between states, leading to heat production. The aggregated absorption of a photosensitizer differs from monomeric absorption; binding to macromolecules shifts absorption, extends the lifetime of the triplet state, and reduces aggregation [39].

So far, few PSs have been developed and approved for clinical practice; several PSs are undergoing clinical trials. The most common clinically used photosensitizers are presented in Table 1.

Photofrin^®^ is a photosensitizer that has been used for years by medical institutions around the world for the treatment of cancer. Its effectiveness and safety have gained wide recognition. Nevertheless, Photofrin^®^ has several noticeable drawbacks [40]. These include dark cytotoxicity, skin phototoxicity, a complex oligomeric composition, and a limited absorption range. One significant challenge arises from the fact that its wavelength of excitation is 630 nm, which is shorter than the ideal range of light transmission through tissues. As a consequence, this limits the depth of the PS excitation during the procedure. Moreover, the drug may remain on the skin for several weeks, which increases the likelihood of side effects related to skin photosensitivity.

Ameluz^®^ is a preparation containing an endogenous metabolite in the form of 5-aminolevulinic acid (5-ALA), in the form of a gel based on a nanoemulsion. It is registered in the European Union (EU) for the PDT of mild-to-moderate actinic keratosis and scalp alopecia [41]. 5-aminolevulinic acid occurs in all eukaryotic cells, where it serves as a precursor for the synthesis of protoporphyrin IX (PpIX) [42].

AlaCare^®^ is a self-adhesive patch containing 5-aminolevulinic acid that is intended for use on the skin and has been developed for use in the photodynamic therapy of mild-to-moderate actinic keratosis [43].

Levulan^®^ is a natural precursor of porphyrins which is metabolized to protoporphyrin IX (Pp IX) in vivo [44].

Hexvix^®^ (hexaminolevulinate) is a stronger lipophilic derivative, which is an improved ALA ester. After intravesical administration, hexaminolevulinate, as compared to 5-ALA, has deeper penetration of the urinary tract epithelium and achieves higher levels of PpIX at much lower concentrations [45]. Protoporphyrins synthesized by hexaminolevulinate administration are generally called “photoactive porphyrins”. Unlike 5-ALA, hexaminolevulinate cannot be administered systemically, only topically.

Foscan^®^ is a photosensitizing agent also known as temoporfin. In the EU, it was approved in October 2001 as an alternative treatment to chemotherapy and radiotherapy in patients with advanced squamous cell carcinoma of the head and neck [46].

Talaporfin, also known as aspartyl chlorine, mono-L-aspartyl chlorine e6, NPe6, or LS11, is a chlorin-based photosensitizer used in PDT. This PS absorbs red light with a wavelength of 664–667 nm that is delivered by a laser which is specially tuned for this range. Talaporfin was approved in Japan in 2004 for the PDT treatment of lung cancer and was marketed under the name Laserphyrin [47,48].

Metvix^®^, or methyl aminolevulinate, is approved in the EU for use in PDT in the treatment of sBCC, nBCC, and thin or non-hyperkeratotic and non-pigmented AK on the face and scalp. Random observations of surface fluorescence three hours after the application of a photosensitizer can be used to detect cancer and determine its boundaries [49].

Verteporfin, a porphyrin derivative, has strong photosensitizing properties that cover all of the basic requirements for the effective PDT of ocular neovascularization [50]. Observations suggest that the mode of action of verteporfin therapy is to cause damage within blood vessels, resulting in the formation of blood cells and the targeted occlusion of specific blood vessels. Verteporfin photodynamic therapy operates at an appropriate power output, making it suitable for a variety of applications that are similar to those for which chemotherapeutic agents are used [51].

TOOKAD^®^ Soluble, developed by Salomon, Scherz, and colleagues in Israel, is the world’s first approved, palladium-based photosensitizer for the treatment of low-risk prostate cancer using PDT vascular targeted therapy (VTP). Derived from Bacteriochlorophyll α (Bchl), it is a negatively charged molecule resembling their earlier PS WST09. Unlike WST09, TOOKAD^®^ Soluble avoids cardiovascular problems and liver toxicity. It forms a non-covalent complex with the human serum albumin, remaining in the plasma until removal, making it an ideal choice for VTP [52,53].

A search of the literature shows that many aspects of fiber optics are unexplored for use in photooxidation chemistry and that there is much that can be learned by applying a sensitizer and singlet-oxygen delivery device to treat cancer. Cancers located proximal to vital tissue are expected to be treated by the proposed ^1^O_2_ delivery device. The mechanistic understanding of the adsorption process and of the sensitization properties of sensitizer with visible light and optimized ^1^O_2_ formation and diffusion into the surrounding biological solution needs to be further expanded [54,55]. Porous Vycor glass can be used [56], and provides a number of advantages with respect to the proposed application. Interconnected pores and channels permit gases to be transported through the PVG. The available silanol groups provide reaction sites to covalently anchor the sensitizers to the silica matrix in adequate numbers to efficiently adsorb the radiation. Although porous, these materials possess a high structural integrity.

One direction of research into next-generation PDTs is to chemically attach PS dyes that are approved for clinical use to up-conversion nanoparticles (UCN). The dyes absorb visible light emitted from the up-conversion of near-infrared light (NIR) to visible light after the nanoparticle is irradiated with NIR, which greatly extends the treatment depth. This is a relatively new field of research and, to date, few examples of up-conversion nanoparticle-PS constructs have been developed and further research is needed to realize their full potential [57,58,59,60,61,62,63,64,65,66].

There are several probes that can be utilized to detect and quantify the amount of ^1^O_2_ generated by a PS in solution and in cells that rely on absorption decay or increasing probe fluorescence emission. Fluorescence probes are useful for confirming the presence of ^1^O_2_ and approximating its concentration. Real-time dosimetry is more challenging and requires the direct measurement of ^1^O_2_ luminescence at 1270 nm. Singlet oxygen sensor green (SOSG or trans-1-(2′-methoxyvinyl) pyrene) generates chemiluminescence upon decomposition to 1-pyrenecarboxaldehyde at 465 nm. Singlet oxygen sensor green is cell-impermeant and can be used either in the presence of model cells or in aqueous solution to approximate the concentrations of generated extracellular ^1^O_2_.

The cell-permeable dye 2′,7′-dichlorofluorescin diacetate (DCFDA) can be used to confirm the presence of ROS intracellularly [67]. Fluorescence of the oxidized products of DCFDA confirms the presence of ROS non-specifically and would not be a specific test for the presence of ^1^O_2_. The cell-permeable dye 2′,7′-dichlorofluorescin diacetate (DCFDA) is deacetylated inside the cell to a non-fluorescent compound which reacts with ROS to form 2′,7′-dichlorofluorescin (DCF), which can be detected by flow cytometry by excitation at 495 nm and emission at 529 nm.

#### 1.2.3. Light

Selectivity for treating tumor cells and the sparing of healthy cells is a major challenge. Light is the third necessary component to perform PDT. The concept of light-induced cell death through chemical interactions was recognized a hundred years ago. In 1900, O. Raab described cell death caused by light and chemicals [67].

Various light sources are used for modern PDT, including lasers, incandescent light, and light-emitting diodes [68]. Laser sources are expensive and require optical systems for broader tissue irradiation, while non-laser sources, such as conventional lamps, can be combined with optical fibers to control the wavelength of light during tissue treatment. However, conventional lamps can cause unwanted thermal effects, which is a problem in PDT. Alternatively, LEDs are cost-effective, safer, thermally non-destructive, and available in flexible systems [69]. Another suitable light source for PDT is natural light, the use of which is called daylight PDT. Daylight PDT replaces artificial light with natural sunlight to treat skin lesions such as actinic keratosis. Daylight PDT offers benefits such as minimal patient discomfort and shorter clinical visits, as patients can complete the therapy at home. Moreover, in the treatment of actinic keratosis, daylight PDT appears to be as effective as traditional PDT [70].

The penetration of light into cancer tissue is complicated and involves the processes of reflection, scattering, and absorption, which are influenced by the type of tissue and the wavelength of the light [71]. Tissues contain endogenous chromophores such as hemoglobin, myoglobin, and cytochromes, which can interfere with the photodynamic process by competing with the PS for light absorption [71,72]. Longer wavelengths, such as red light, penetrate tissue more effectively, usually within the “tissue optical window” of 600–1200 nm [8]. Shorter wavelengths (<600 nm) penetrate less effectively, causing increased skin sensitivity to light, while longer wavelengths (>850 nm) lack the energy needed to generate ^1^O_2_. Therefore, the practical “phototherapeutic window” for PDT ranges between 600 and 850 nm [11,73]. The effectiveness of PDT depends on the precise delivery of light to the target tissue and factors such as the light fluence, light fluence ratio, light exposure time, and method of light delivery (single or fractionated). Light fluence measures the total energy of the exposed light per unit area (J/cm^2^), while the light fluence coefficient represents the energy incident per second per unit area (W/cm^2^) [74,75].

Deeper tissue PDTs with long-wavelength absorption of PS along with selective localization in diseased tissue are needed. The preferred PSs for deep tissue PDT are red-absorbers for the formation of ROS such as singlet oxygen. Advances in PDT cancer treatments may be realized with the use of nanoparticles that can absorb NIR and emit visible light. The conversion of NIR to visible light by lanthanide nanoparticles is a process known as up-conversion, as previously described. A dye used in PDT must absorb visible light to initiate anti-cancer effects. Visible light can only penetrate through several millimeters of tissue, greatly limiting the tissue-depth range of current treatments. NIR light may penetrate several centimeters into tissue.

## 2. PDT Photophysical Processes

**Figure 2:**
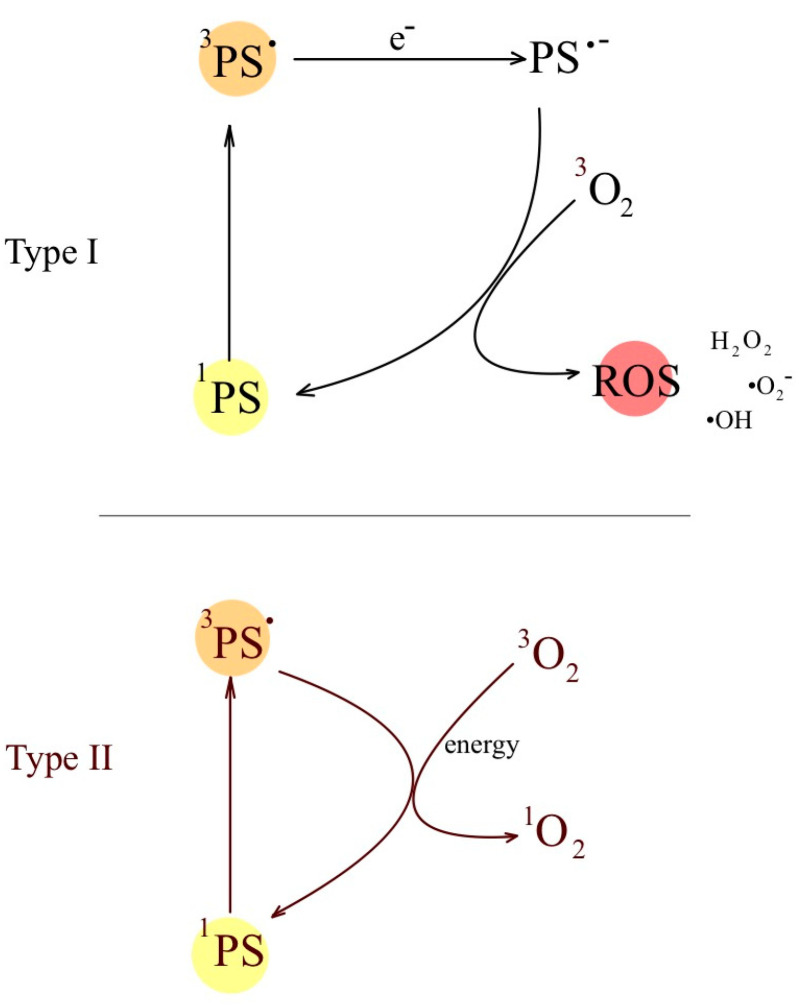
Two main types of photochemical reactions in target cells: Type I reactions and Type II reactions. A Type I reaction involves electron transfer by the excited photosensitizer, leading to the formation of reactive free radicals that cause cell damage. The Type II reaction involves formation of singlet oxygen (^1^O_2_) that reacts with biomolecules such as lipids, proteins, and DNA, leading to oxidative damage and cell destruction.

The photophysical processes of PDT involve interactions between the PS molecule and light during the treatment. These processes involve the absorption, emission, and energy transfer of the photosensitizer as it passes through different electronic states (Table 2). Understanding the photophysical responses of PDT helps to select appropriate photosensitizers, optimize light parameters, and design effective therapeutic strategies to maximize the therapeutic effect while minimizing the damage to healthy tissues. This is crucial to optimizing the effectiveness of PDT. PDT photophysical processes are based on the absorption of light by a PS molecule, which is the first step in the described therapy. Light energy excites the PS from the ground state to the first excited singlet state (S1). For the generation of ROS, the photosensitizer must intersystem cross to the excited triplet (T1) state. Photosensitizer molecules in the excited singlet state (S1) release excess energy in the form of fluorescence or heat. The light emitted by fluorescence has less energy (longer wavelength) compared to the light that is absorbed. This emission does not contribute to the PDT effect, but can be used for diagnostic purposes to visualize the degradation of the photosensitizer. In some cases, instead of emitting fluorescence, the photosensitizer undergoes intersystem crossing (ISC), a process in which it changes from an excited singlet state (S1) to an excited triplet state (T1). The T1 state is relatively long-lived compared to the singlet state, which is critical for generating ROS. Photosensitizers in the excited triplet state (T1) can collide and transfer energy to molecular oxygen (^3^O_2_) in the ground state. This interaction leads to energy transfer, resulting in the formation of cytotoxic singlet oxygen (^1^O_2_). Triplet-state PSs undergo Type I and Type II reactions (Figure 2). The Type I reaction involves electron transfer between the excited photosensitizer and ^3^O_2_ or other molecules, which leads to the formation of reactive free radicals that cause cell damage. The Type II reaction involves the direct formation of ^1^O_2_. Singlet oxygen reacts electrophilically with biomolecules such as lipids, proteins, and DNA, leading to oxidative damage and cell destruction. Cell death occurs as a result of the formation of cytotoxic ROS, especially singlet oxygen (Type II) and hydroxy radical (Type II), which causes damage to cellular structures and processes, ultimately leading to cell death through apoptosis or necrosis.

Efforts by Muhr et al., Hahn et al., and Busch et al., advanced the parameters for PDT reactive oxygen species modeling and explicit PDT modeling [76,77,78]. For example, about singlet oxygen molecules are required to kill each tumor cell when using benzoporphyrin derivative (BPD) as the PS (72).

### 2.1. Type I and Type II Reactions

PDT involves two main types of photochemical reactions, known as Type I and Type II reactions [79]. These reactions are responsible for the production of cytotoxic ROS, which ultimately leads to the destruction of target cells or microorganisms.

In a Type I reaction (Table 3), the photosensitizer undergoes electron transfer reactions upon excitation. This process can lead to the formation of reactive free radicals, such as superoxide radicals (O_2_^•−^) and hydroxyl radicals (•OH), which cause damage to cellular structures, including proteins, lipids, and DNA.

In the Type II reaction (Table 4), the photosensitizer, being in the triplet state, reacts directly with ^3^O_2_ in the ground state. This interaction produces highly reactive ^1^O_2_, which is a highly cytotoxic compound. Singlet oxygen is a highly oxidizing molecule that can damage cellular components including lipids, proteins, and DNA, leading to cell death.

Both Type I and Type II reactions contribute to the therapeutic effects of PDT. The specific contribution of each type of reaction depends on the type of photosensitizer used and the local microenvironment. Understanding the balance between Type I and Type II responses is essential to optimizing the effectiveness of PDT in various medical applications.

### 2.2. Jabłoński’s Diagram

**Figure 3:**
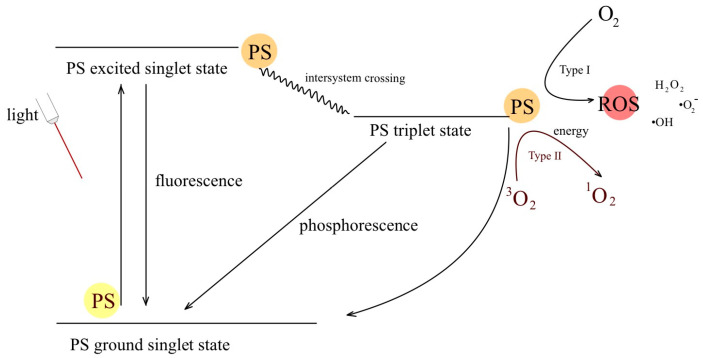
Jabłoński diagram showing photophysical processes occurring during PDT.

The Jabłoński diagram is a graphical representation used to explain the photophysical processes that occur during PDT and other photochemical reactions (Figure 3). Its name comes from the Polish physicist Aleksander Jabłoński, who first introduced it in the 1930s. The Jabłoński diagram consists of energy levels and transitions that describe the behavior of molecules that absorb a photon. It helps illustrate the various electronic states and energy changes associated with a photochemical reaction. Key elements of the Jabłoński diagram in PDT are:

1. Ground State (S0): The photosensitizer molecule in its lowest energy state, called the ground state prior to excitation;
2. Excited states (S1, S2, etc.): When a photosensitizer absorbs a photon of light, it is promoted to higher energy states known as excited singlet states (S1, S2 etc.). These excited states are transient and relatively short-lived;
3. Singlet state (S1): The first excited singlet state (S1) is of particular importance in PDT. From this state, the photosensitizer can undergo various processes:
  A. Fluorescence (F): Some photosensitizer molecules return to the ground state (S0) by emitting fluorescence, where they release excess energy in the form of lower-energy photons of light;
  B. Radiation-free relaxation: Other photosensitizer molecules lose energy in radiation-free processes that do not involve the emission of photons;
4. Intersystem crossing: Photosensitizer molecules in the excited singlet state (S1) can undergo ISC to the longer-lived excited triplet state (T1). This is an essential step in PDT because the triplet state is the state responsible for the production of reactive oxygen species (ROS);
5. Triplet state (T1): In the excited triplet state (T1), the photosensitizer can interact with molecular oxygen (O_2_) in the ground state, leading to the production of singlet oxygen (^1^O_2_), a highly reactive and cytotoxic species;
6. Type I and Type II reactions.
  A. Type I reaction involves electron transfer from the photosensitizer, which leads to the formation of reactive free radicals that can damage cellular structures;
  B. Type II reaction: Singlet oxygen is generated and reacts directly with biomolecules such as lipids, proteins, and DNA, causing oxidative damage and ultimately leading to cell death.

The Jabłoński diagram provides a visual representation of these complex photophysical processes, helping scientists and practitioners to understand and optimize the PDT process for specific medical applications. It has become an essential tool for research on the mechanisms behind PDT and other photochemical reactions.

## 3. Mechanism of Cell Death Induced by PDT

**Figure 4:**
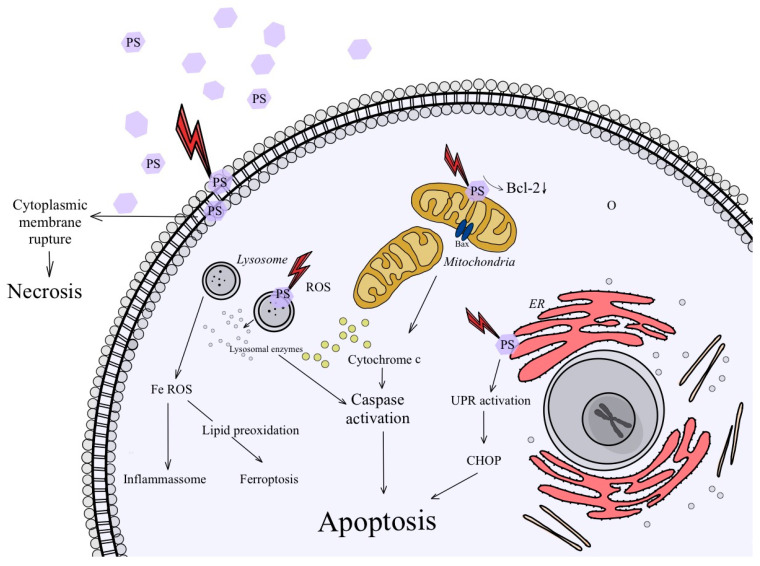
Schematic representation of cancer cell death by apoptosis and necrosis after PDT activation.

PDT can lead to cell death through several different mechanisms, depending on the specific conditions and target cells involved. The main mechanisms of cell death in PDT include apoptosis, necrosis, and autophagy [80,81,82] (Figure 4).

It should be noted that the specific cell death mechanisms activated after PDT may vary depending on the type of photosensitizer used, the light dose, the tumor microenvironment, and the sensitivity of the target cells [83,84].

### 3.1. Apoptosis

Apoptosis is a natural process triggered by specific stimuli that induce distinct morphological changes in cells, such as reducing their volume, chromatin condensation, nuclear rounding, membrane blebbing, and others [85,86]. This process involves the activation of enzymes called caspases and endonucleases [87]. Apoptosis can be divided into intrinsic and extrinsic pathways, each resulting from different factors. Intrinsic apoptosis is a regulated form of cell death caused by various disturbances in the cell microenvironment, such as DNA damage, the presence of reactive oxygen species (ROS), endoplasmic reticulum stress, and growth factor withdrawal [88]. It is initiated independently of cell-surface receptors and involves the cleavage of a protein called Bid. Bid translocates to the mitochondria and activates Bax and Bak proteins, which leads to mitochondrial permeability and the release of cytochrome c. This in turn triggers the activation of Apaf-1 and procaspase 9, ultimately leading to the activation of caspase 3/7 and programmed cell death [89,90]. On the other hand, the extrinsic pathway involves signaling via a transmembrane receptor. It begins with the activation of FADD, which recruits pro-caspase 8. This leads to the activation of caspase 8 and then caspase 3, which ends in apoptosis [91]. The induction of apoptosis after PDT depends significantly on the intracellular localization of the photosensitizer (PS). For example, when the PS localizes to the lysosome, PDT causes the destruction of the lysosomal membrane, leading to the release of cathepsins and proteases into the cytosol. This, in turn, triggers the cleavage of Bid into truncated Bid (tBid), a pro-apoptotic protein [92].

### 3.2. Autophagy

Autophagy is a cellular recycling process in which cells engulf and enclose organelles and cytosol in vacuoles called autophagosomes. Although autophagy typically functions as a means of cell self-preservation, it may also play a role in increasing cell death, particularly in cells lacking apoptosis [93,94]. In the context of PDT, autophagy can lead to cell survival or death, depending on the intensity of the cytotoxicity during treatment. It may help recycle damaged mitochondria or endoplasmic reticulums before triggering apoptosis. However, when PDT cytotoxicity is significant, both apoptotic and autophagic pathways can lead to cell death. Additionally, autophagy is observed in cells that are resistant to apoptosis [95].

### 3.3. Necrosis

Necrosis represents another type of cell death resulting from severe cell damage that compromises the integrity of the cell membrane [87]. The consequence of necrosis is the leakage of cellular contents into the surrounding tissues, which triggers an inflammatory reaction [96]. Necrotic cells often undergo oncosis, which leads to cell rupture. Unlike apoptosis, necrosis is an energy-independent form of cell death in which cells experience severe damage due to sudden shocks such as radiation, heat, chemicals, or hypoxia. For a long time, necrosis was believed to be the only mode of cell death after PDT treatment, especially when observed in the terminal state of cells in vivo. The presence of cellular fragments, which were attributed to late apoptotic cells, were thought to be the result of necrotic cell death.

However, recent years have brought a deeper understanding of the mechanisms of cell death, leading to a more comprehensive classification of various cell-death pathways beyond apoptosis and necrosis. This broader perspective has opened up opportunities to improve the effectiveness of PDT through combination therapies.

## 4. Anticancer Properties of Singlet Oxygen

In the PDT mechanism, main roles are played by reactive oxygen species (ROS) and reactive nitrogen species (RNS), which are of key importance in the mechanism of action of PDT. In PDT, a photosensitizing agent is activated by light to produce ROS, including singlet oxygen (^1^O_2_), which leads to the selective destruction of target cells. Various aspects related to ROS and RNS in cancer cells, including protective mechanisms, are used to oppose apoptosis-inducing signals. In the context of PDT, the formation of ROS, including singlet oxygen, plays a key role in initiating apoptosis in target cells. Therefore, understanding how cancer cells protect themselves against oxidative and nitrosative stress is important to appreciate the challenges facing the effective use of PDT in cancer treatment. The complex interaction between ROS and RNS has a significant impact on cell signaling and apoptosis, which are important aspects of the mechanism of action of PDT on cancer cells.

**Figure 5:**
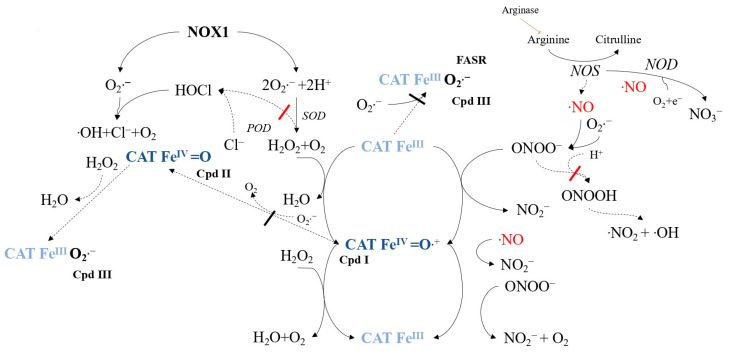
The protective mechanism in tumor cells against intercellular reactive oxygen species/reactive nitrogen species (ROS/RNS)-induced apoptosis signaling involves various components. These include NADPH oxidase-1 (NOX1), superoxide dismutase (SOD), catalase (CAT), and the FAS receptor (FASR), which is a member of the tumor necrosis factor receptor family. These proteins are found on the cell membrane, while peroxidase (POD), formerly associated with membrane-bound dual oxidase (DUOX), has been released into the extracellular space. Arginase, NO synthase (NOS), and NO dioxygenase (NOD) are located intracellularly. Catalase can form an active intermediate compound I (Cpd I) during its reaction with substrates, while compound II (Cpd II) and compound III (Cpd III) remain inactive. This figure illustrates catalase’s protective role in the HOCl and NO/peroxynitrite signaling pathways and highlights the modulatory effect of SOD in preventing superoxide anion-dependent inhibition of catalase.

**Figure 6:**
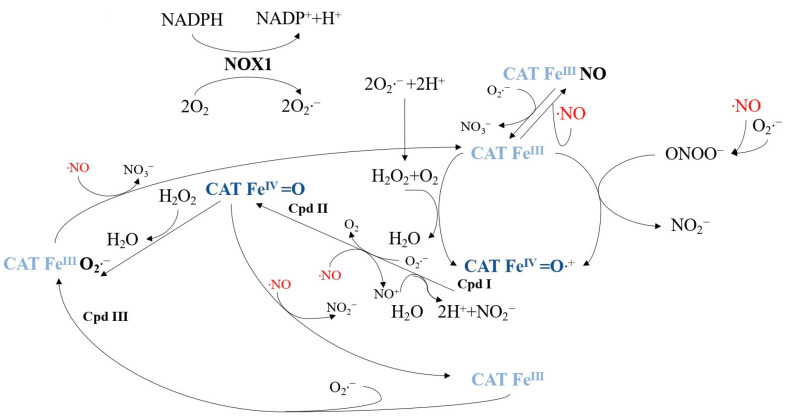
This figure represents membrane-associated catalase in tumor cells, a pivotal enzyme at the intersection of reactive oxygen species (ROS) and reactive nitrogen species (RNS). It provides an overview of the interactions involving H_2_O_2_, peroxynitrite, NO, and superoxide anions with catalase and its intermediates. Alongside compound I (Cpd I), compound II (Cpd II), and compound III (Cpd III) of catalase, defined in Figure 1, this figure also portrays the inactive complex formed between catalase and NO (CAT FeIII•NO).

Tumor progression relies on membrane-associated NADPH oxidase 1 (NOX1) generating extracellular superoxide anions and H_2_O_2_ (Figure 5), which are crucial for maintaining malignant cell growth [97,98]. However, tumor cells must protect against apoptosis-inducing HOCl and NO/peroxynitrite signaling [99,100,101,102]. HOCl signaling involves the synthesis of H_2_O_2_-dependent HOCl by dual oxidase (DUOX), triggering apoptosis through hydroxyl radical formation [103,104]. NO/peroxynitrite signaling leads to peroxynitrite formation and subsequent apoptosis via hydroxyl radicals [105,106]. To resist exogenous H_2_O_2_ [107,108] and intercellular signaling [109], tumor cells employ a protective system comprising membrane-associated catalase (CATFeIII) to decompose H_2_O_2_ and oxidize NO and peroxynitrite [75,76,110,111]. This process involves compound I (CAT-FeIV=O•+) [77,112]. Superoxide anions inhibit catalase [113,114,115], so tumor cells express membrane-associated superoxide dismutase (SOD) to maintain catalase activity [81,115]. Figure 6 illustrates catalase’s role in controlling the ROS/RNS balance, with NO influencing the catalase activity [110].

The interplay between SOD/catalase and active NOX1 in tumor cells enables selective apoptosis induction when the protective system is disrupted, offering the potential for selective tumor cell apoptosis in vitro and in vivo. Singlet oxygen, a versatile member of the ROS family [116], has significant potential for controlling oncogenesis and antitumor therapies. It can deactivate catalase by reacting with histidine at its active center [117], thus disrupting the antioxidant defense of tumor cells.

**Figure 7:**
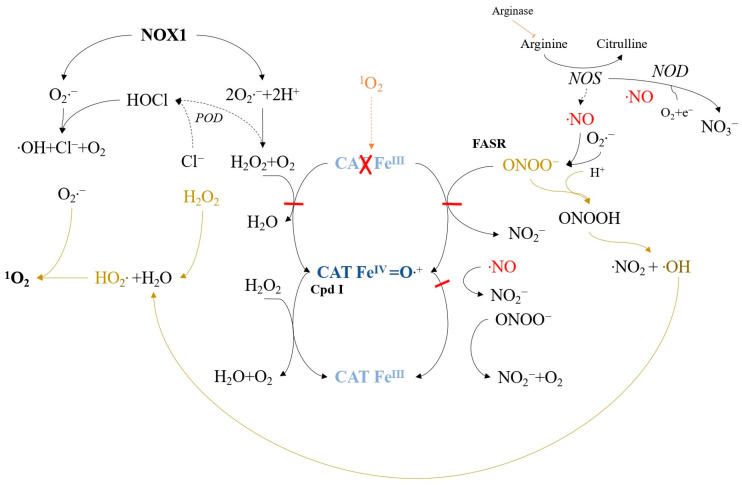
Catalase inactivation due to low concentrations of singlet oxygen is described as an autoamplificatory mechanism driven by FAS receptor (FASR)-mediated enhancement of NADPH oxidase-1 (NOX1) and NO synthase (NOS) activities, resulting in secondary singlet oxygen production. The FASR belongs to the tumor necrosis factor receptor family. Low levels of exogenous singlet oxygen locally deactivate membrane-associated catalase, leading to interactions between H_2_O_2_ and hydroxyl radicals from peroxynitrite, ultimately generating hydroperoxyl radicals and singlet oxygen (^1^O_2_).

Experimental models have demonstrated that low concentrations of extracellular singlet oxygen, generated by illuminating the photosensitizer photofrin, locally deactivate catalase on tumor cell membranes (Figure 7) [118]. This inactivation leads to the accumulation of H_2_O_2_ and peroxynitrite (generated outside tumor cells) and prevents NO oxidation. Proton pumps cause peroxynitrite protonation, leading to peroxynitrous acid formation and subsequent •NO_2_ and hydroxyl radical production.

The interaction between H_2_O_2_ and hydroxyl radicals generates hydroperoxide radicals, which can react with NOX1-derived superoxide anions to form ^1^O_2_. This sequence of reactions reconciles previous studies [119,120] and supports singlet oxygen generation through H_2_O_2_ and peroxynitrite interaction involving hydroxyl and hydroperoxide radicals.

**Figure 8:**
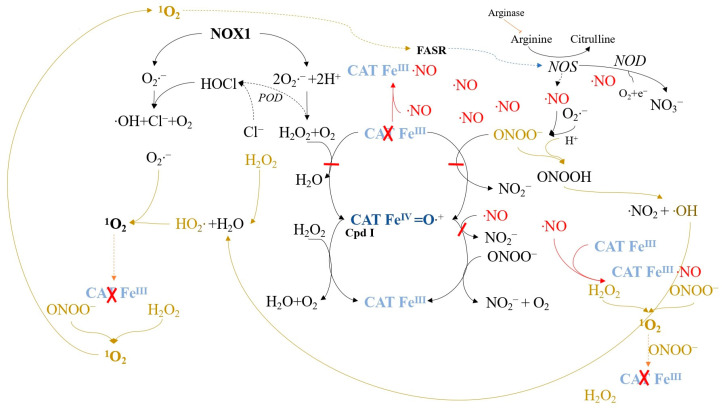
Secondary singlet oxygen, produced through the reactions described in Figure 7, activates the FASR, subsequently enhancing NOX1 activity and inducing NOS expression, contributing to increased singlet oxygen generation and catalase inactivation.

Secondary ^1^O_2_ generated after catalase inactivation can further inactivate catalase or activate the FAS receptor (FASR) [121] (Figure 8). FAS receptor activation enhances NOX1 activity and induces NO synthase (NOS) expression [122], leading to increased superoxide anions, H_2_O_2_, NO, and peroxynitrite. This mixture generates secondary singlet oxygen, followed by catalase inactivation, enabling the reactivation of intercellular ROS/RNS-mediated apoptosis signaling. The increase in local NO during this process may also inhibit catalase reversibly at other sites, leading to additional secondary ^1^O_2_ generation.

**Figure 9:**
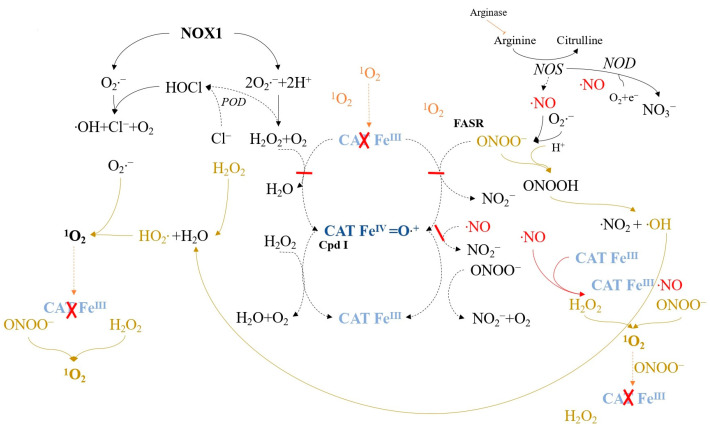
Catalase inactivation by high concentrations of ^1^O_2_ follows an auto-amplificatory mechanism based on the generation of secondary ^1^O_2_. Elevated levels of exogenous ^1^O_2_ cause multiple instances of local catalase inactivation on tumor cell membranes. This facilitates optimal ^1^O_2_ generation through complex interactions between H_2_O_2_ and peroxynitrite, followed by catalase inactivation, thereby reactivating intercellular reactive oxygen species (ROS)-dependent apoptosis signaling.

When applying relatively high concentrations of exogenous ^1^O_2_ (Figure 9), secondary extracellular ^1^O_2_ generation is necessary for the optimal catalase inactivation and apoptosis-inducing intercellular ROS/RNS signaling of tumor cells. Under these conditions, FAS receptor amplification is not required, and catalase inactivation and signaling occur even when FAS receptor or caspase-8 functions are disrupted [118].

**Figure 10:**
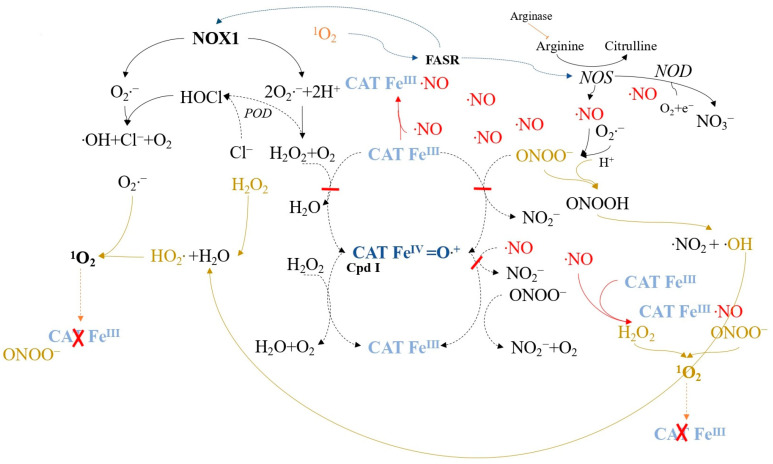
Direct activation of the FAS receptor (FASR), a member of the tumor necrosis factor receptor family, by singlet oxygen or its ligand initiates singlet oxygen-mediated catalase inactivation. FASR activation by ^1^O_2_ leads to NOX1 activation and NOS expression induction. Elevated free NO levels reversibly inhibit catalase, interfering with reactions and facilitating hydroperoxide radical formation, singlet oxygen generation, and catalase inactivation. Ultimately, this process reactivates intercellular reactive oxygen species/reactive nitrogen species (ROS/RNS)-dependent apoptosis signaling.

**Figure 11:**
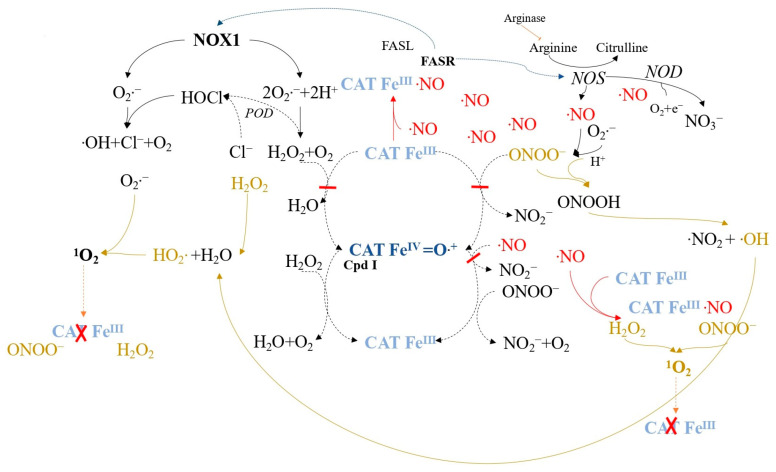
Activation of the FASR by its ligand triggers the same sequence of events as depicted in Figure 10 for FASR activation by ^1^O_2_.

Low concentrations of ^1^O_2_ are more likely to initially target catalase, which is abundant on the membrane, although FAS receptor activation can also lead to the same outcome by inducing NOS and increasing NO (Figure 10). This sets off a cascade of secondary ^1^O_2_ generation, catalase inactivation, and the reactivation of intercellular ROS/RNS-mediated apoptosis signaling. The feasibility of this FAS receptor-dependent pathway has been confirmed, as it results in singlet oxygen generation, catalase inactivation, and apoptosis signaling reactivation (Figure 11) [100]. However, NOX1 activity enhancement through FAS receptor activation alone is insufficient for catalase inactivation and signaling reactivation [100,123].

Increasing the endogenous NO concentration through methods like arginase inhibition, arginine addition, or NOD inhibition causes reversible catalase inhibition on tumor cell surfaces, leading to extracellular secondary singlet oxygen formation and FAS receptor activation. This process amplifies singlet oxygen generation, enabling subsequent intercellular ROS/RNS signaling and mitochondrial apoptosis induction. Inhibiting NOD by secondary plant compounds provides insight into tumor prevention and novel antitumor drugs [100,104,124].

In this section, we explore the modulation of NO metabolism and the generation of ^1^O_2_ in the context of tumor therapy. Increased endogenous NO levels, achieved through various means like arginase inhibition, arginine addition, or NOS induction by interferons (not shown), as well as the inhibition of NO dioxygenase (NOD) by compounds including anthocyanidins, flavonoids, antifungal azoles, diallyldisulfide, artemisinine, and taxol, lead to localized reversible catalase inhibition on tumor cell surfaces [104]. This inhibition triggers extracellular secondary singlet oxygen formation and amplifies singlet oxygen generation via the singlet oxygen-dependent activation of the FAS receptor. The subsequent inactivation of a sufficient concentration of membrane-associated catalase allows intercellular ROS/RNS signaling and induction of the mitochondrial pathway of apoptosis. In particular, the inhibition of NOD by various secondary plant compounds [104] offers insights into the tumor-preventive effect of these agents and hints at novel antitumor drug development possibilities. The selective apoptosis induction in tumor cells through NO-mediated catalase inhibition and singlet oxygen action opens doors to the potential optimization of photodynamic therapy, suggesting the development of cell-impermeable photosensitizers targeting tumor cells via singlet oxygen-dependent catalase inactivation. Cold atmospheric plasma (CAP) generates biologically active ROS/RNS [125,126], while plasma-activated medium (PAM) demonstrates selective antitumor effects [127,128,129], potentially driven by a peroxynitrite-mediated mechanism involving nitrite and H_2_O_2_ [130,131]. Both CAP and PAM leverage ^1^O_2_ in rational tumor therapy with ROS/RNS signaling selectivity [132]. Salicylic acid and its derivatives directly inhibit catalase, facilitating the reactivation of intercellular ROS/RNS signaling, independent of singlet oxygen action. Combining *N*-acetyl salicylic acid with NO donors reveals a synergistic effect which targets membrane-associated catalase via ^1^O_2_. Nitric oxide-donating *N*-acetyl salicylic acid shows promise in preventing pancreatic cancer, emphasizing the potential of ^1^O_2_ and catalase as antitumor targets [133].

PDT Arguments [134,135,136]:

- The accumulation of research work in recent years suggests that PDT is a promising therapeutic alternative in cancer research;
- The PDT methodology allows for the manual delivery of a PS and manual irradiation, thus minimizing the damage to healthy tissue;
- A PS that is directly delivered to the sick tissue does not cause side effects on healthy tissue;
- PDT is less invasive than surgery and allows for quick and simple application;
- The low risk profile of minimal side effects and tissue resistance allows for repeated applications in the same place;
- Both older people and people that are too sick to undergo surgery can be treated by PDT;
- The use of optical fibers and a wide range of wavelengths allows for both intra-tissue and endoscopic applications covering a wide range of tissue applications;
- The nuclear localization of the PS results in a low risk of additional radical-generated DNA mutations after treatment;
- Immune sensitization: Surgery, chemotherapy, and radiotherapy have immunosuppressive effects, while ROS produced by PDT induce vascular damage, leading to thrombosis, hemorrhage, and inflammation. This alerts anti-tumor immunity and thus sensitizes the immune system to additive attacks on the tumor microenvironment of surviving cells.

To the best of our knowledge, understanding the relationship between tumor size and PDT efficacy will be useful to develop new photosensitizers for PDT. Therefore, the use of a PS and light doses to generate ^1^O_2_ can be dependent on the tumor volume and localization. Tumor responses after PDT treatment in relation to tumor size were already discussed in the literature [137,138,139,140,141]. PDT is highly influenced by an efficient generation of ^1^O_2_. Thus, a large quantity of ^1^O_2_ is needed for the enhanced therapeutic efficiency of PSs in PDT [141,142]. Because of the short lifetime of ^1^O_2_ (about 200 ns) produced by PDT in cells, and the limitation of the diffusion distance (about 50 nm), only the organelles close to ^1^O_2_ will receive oxidative damage. Therefore, the localization of PSs in important subcellular organelles such as the nucleus, mitochondria, and endoplasmic reticulum can enhance the phototoxicity of ^1^O_2_ and enhance the efficacy of PDT [142].

The results of PDT clinical research done by Furukawa et al. showed that central-type early stage lung cancer < 1.0 cm in diameter shows almost a 100% complete response to PDT [138]. In the group of patients with lesions > or = 1.0 cm, the complete response and 5-year survival rates were 58.1% and 59.3% [138].

Usuda et al. reported that mono-l-aspartyl chlorine e6 (NPe6, Laserphyrin), a second-generation photosensitizer with a lower photosensitivity than Photofrin (porfimer sodium), was approved by the Japanese government, and a phase II clinical study using NPe6 with a new diode laser demonstrated an excellent antitumor effect and low skin photosensitivity [139]. In addition, one of the recommended criteria for the treatment of lung cancer using PDT is a tumor size less than 1 cm in the greatest diameter [139].

Also, Usuda et al. discussed that most centrally located early lung cancers < 1.0 cm in diameter do not invade beyond the bronchial cartilage, and PDT with Photofrin is currently recommended as a treatment option for such lesions [140].

When the esophageal cancers’ tumor size was up to 1 cm, PDT resulted in complete regression in 100% of patients. However, when the tumor size is 3.1–5.0 cm, only 33.3% of patients achieve complete regression [141].

## 5. Summary

The recent studies related to PDT on the cellular level are presented here. We summarize the various chemical reactions that contribute to cellular PDT which results in the production of ROS, with ^1^O_2_ being of great importance. Singlet oxygen generated in diseased tissue does not cause side effects in healthy tissue. The effectiveness of PDT is due to the short-lived and highly reactive nature of ROS, affecting mainly cells located in close proximity to the PS, in turn causing local tissue destruction. PDT is less invasive than surgery and allows for quick and simple application. Furthermore, PDT can stimulate the immune response, further enhancing target cell destruction and potentially offering long-term benefits. Tumor size and volume are important in order to obtain PDT therapeutic effects.

## Figures and Tables

**Table 1:** Photosensitizers used in clinical PDT, with trade name, chemical name, molecular formula, manufacturer, and main applications.

Trade Name of Photosensitizer	Chemical Group	Chemical Name of the Photosensitizer	Molecular Formula	Producer	Application
Photofrin^®^	Porphyrin	Porfimer sodium	C_34_H_38_N_4_NaO_5_+	Axcan Pharma, Quebec, QC, Canada	Esophageal, lung and bronchial cancer, bladder cancer, stomach cancer
Ameluz^®^	Porphyrin	aminolevulinic acid hydrochloride	C_5_H_9_NO_3_•HCl	DUSA, Wilmington, MA, USA	Actinic keratosis and basal cell carcinoma
AlaCare^®^	Porphyrin	5-aminolevulinic acid	C_5_H_9_NO_3_	photonamic GmbH und Co. KG, Pinneberg, Germany	Actinic keratosis and basal cell carcinoma
Levulan^®^, 5-ALA	PpIX precursor	5-aminolevulinic acid	C_5_H_9_NO_3_	DUSA	Actinic keratosis and basal cell carcinoma
Hexvix^®^, HLA	PpIX precursor	Hexaminolevulinate	C_11_H_21_NO3	Photocure, Oslo, Norway	Bladder cancer diagnosis
Foscan^®^, mTHPC	Chlorine	Meta-tetrahydroxy phenyl chlorine Temoporfirin	C_44_H_32_O_4_N_4_	Biolitec, Jena, Germany	Head and neck cancer
Laserphyrin^®^, Npe6	Chlorine	Mono-L-aspartyl chlorine e6 Talaporfin	C_38_H_37_N_5_O_9_	Meiji Seika, Tokyo, Japan	Lung and esophageal cancers and brain tumors
Metvix^®^, MAL	PpIX precursor	Aminolewulinian metylu	C_6_H_11_NO_3_	Galderma, London, UK	Basal cell carcinoma, Bowen’s disease and actinic keratosis
Visudyne^®^, BPD-MA	Chlorine	Benzoporphyrin derivative monoacid Verteporfin	C_82_H_84_N_8_O_16_	Novartis, Basel, Switzerland	Age-related macular degeneration, non-melanoma skin cancer
TOOKAD^®^ Soluble, WST-11	Padeliporfin	Palladium bacteriopheophorbide monolysotaurine	C_37_H_41_K_2_N_5_O_9_PdS	STEBA Biotech, Luxembourg	Prostate cancer

**Table 2:** Photophysical processes of PDT, including interactions between the photosensitizer molecule and light during the treatment. They involve the absorption, emission, and energy transfer of the photosensitizer as it passes through different electronic states.

Process	Description
light absorption	The first step in PDT—light absorption by the photosensitizer molecule
excitation to higher energy states	Light energy raises the photosensitizer from the ground state to the excited singlet state (S1). The photosensitizer then crosses to the triplet (T1) state through intersystem crossing (ISC) processes.
fluorescence emission	Some photosensitizer molecules in the excited singlet state (S1) can release excess energy in the form of fluorescence. Fluorescence is the emission of light with lower energy compared to absorbed light. This emission does not contribute to the PDT effect, but can be used for diagnostic purposes to visualize the degradation of the photosensitizer.
transfer of energy	The formation of cytotoxic oxygen species that lead to cell death.

**Table 3:** Basic steps of Type I reaction in PDT.

Basic Steps of Type I Reaction in PDT:
The photosensitizer absorbs a photon of light and is excited to its higher energy state.
The excited photosensitizer undergoes electron transfer with nearby molecules, which may be oxygen or other biomolecules.
This transfer results in the formation of reactive free radicals, which initiate a chain reaction resulting in cell damage.

**Table 4:** Basic steps of Type II reaction in PDT.

Basic Steps of Type II Reaction in PDT:
The photosensitizer absorbs a photon of light and is promoted to the excited singlet state that intersystem crosses to the excited triplet state.
The photosensitizer in the excited triplet state reacts with molecular oxygen (O_2_ in the ground state to form ^1^O_2_.
Singlet oxygen then diffuses through the cell and reacts with nearby biomolecules, causing cell damage and cell death.
